# Exploring Parental Intentions to Use Digital Tools to Enhance Parent-Child Sexual Communication in Europe: Cross-Sectional Questionnaire Study

**DOI:** 10.2196/75489

**Published:** 2025-10-10

**Authors:** Talia Rose Hubble, Luca Carbone, Laura Vandenbosch, Jaan Toelen, David De Coninck

**Affiliations:** 1 EGA Institute for Women’s Health University College London London United Kingdom; 2 Media Psychology Lab Faculty of Social Sciences KU Leuven Leuven Belgium; 3 KU Leuven Child and Youth Institute Leuven Belgium; 4 Department of Development and Regeneration KU Leuven Leuven Belgium; 5 Centre for Sociological Research Faculty of Social Sciences KU Leuven Leuven Belgium

**Keywords:** sex education, parent-child sexual communication, sexual health, adolescent, puberty

## Abstract

**Background:**

Parent-child communication about sexuality education is critical for safe adolescent sexual development and well-being. Yet, there is evidence that these conversations are often ineffective. Digital tools have therefore emerged as promising interventions that may assist parents in addressing sensitive or difficult topics. However, our understanding of the factors that may motivate parental adoption of these technologies remains limited.

**Objective:**

This study aimed to explore factors associated with European parents’ intentions to use a digital tool designed to support parent-child sexual communication and complement school-based sexuality education. The study was conducted across the United Kingdom, Belgium, and Italy.

**Methods:**

Using the technology acceptance model, we applied structural equation modeling to identify motivators of parents’ *intention to use* the hypothetical app. This included *perceived usefulness* and *perceived ease of use*. *Perceived usefulness* was further analyzed by its subcomponents—*relevance to parenting* and *quality of technology*—through an alternative 3-construct model. Additionally, the associations between demographic characteristics (age, gender, country of residence, and education level) and the latent constructs were assessed.

**Results:**

The study sample included 1296 parents. The explanatory power of the model was *R*^2^=0.47. *Perceived usefulness* was significantly associated with *intention to use* (β=0.67, *P*<.001), while *perceived ease of use* showed no significant association with either *intention to use* (β=–0.01, *P*>.05) or *perceived usefulness* (β=0.10, *P*>.05). An alternative 3-construct analysis revealed that *relevance to parenting* and *quality of technology* were both independently significantly associated with *intention to use* (β=0.09, *P*<.001 and β=0.59, *P*<.001, respectively). Demographic characteristics were also significantly related to the technology acceptance model constructs in the model.

**Conclusions:**

These findings highlight the critical role of perceived usefulness, specifically relevance to parenting needs and the perceived quality of technology, in shaping parental intentions to use digital parent-child sexual communication tools. Developers of educational digital technologies should therefore prioritize high-quality design features to inspire usage. Future research should evaluate real-world digital tools to assess actual usage, long-term engagement, and their effectiveness in enhancing parent-child sexual communication.

## Introduction

### Background

Sexual health encompasses more than the absence of disease; it includes physical, emotional, psychological, and social well-being [1]. As such, sexuality education must go beyond teaching on anatomy, physiology, and the prevention of sexually transmitted infections and unintended pregnancies. Rather, it should also foster positive self-esteem, the ability to engage in healthy relationships, and the skills needed to navigate adult social and sexual experiences safely and confidently [2]. Effective sexuality education must therefore transcend basic and didactic teaching to engage young people in discussions on diverse attitudes, cultural perspectives, and relationship dynamics [3,4]. This should empower young people to explore their emerging identities and meaningfully engage with the world around them [5].

The role of the sexual health educator may be assumed by different figures in a young person’s life: family members, peers, teachers, as well as the wider social and cultural context the child exists within [6-8]. These all act in a network of influence on an adolescent’s sexual development [9]. Here, we explore parents’ perspectives on a hypothetical intergenerational app designed to support parent-child sexual communication. We proposed an app that would be endorsed by schools and align with regional or national school sexuality education curricula. This app would thus serve as both an educational resource for parents and a prompt to initiating parent-child sexual communication [10]. The app’s aim was to enhance young people’s sexual development through parental education, as well as ensure close collaboration between the timing and content of parent and teacher sexuality education. Thus, the app would be integrated into the school curriculum and would unlock access to sexuality education content at appropriate times in the child’s development, aligned with the timing and topics being covered in school.

### Parent-Child Sexuality Communication

Parents are key agents of sexual socialization [8,11]. Recognized as some of the most significant sexual educators, they provide essential information and instill their children with basic values regarding sexual health and well-being [12]. Previous research has examined what constitutes positive and effective parent-child sexual communication. It has been identified that optimal parent-child sexual communication should involve open, supportive, and reciprocal communication [13-15]. Such high-quality parent-child sexual communication appears to support children’s safe sexual development [16,17], reduce their sexual risk-taking [18,19], increase their testing for sexually transmitted infections [20], and improve psychological sexual well-being [21]. This provides evidence that parent-child sexual communication can have a significant positive impact on young people.

There are, however, several barriers to effective parent-child sexual communication. First, it appears that parents often initiate discussions after their child’s sexual debut [22]. This reduces their ability to influence the young person’s behavior. This delay may be because parents perceive their children as too young for sexuality education [23]. Alternatively, it may stem from a desire to protect their child’s perceived innocence by avoiding topics deemed too mature or sexual [24]. Parents may also feel unsure how to initiate parent-child sexual communication and therefore not be able to overcome this initial hurdle [25].

Second, parents appear to overestimate the frequency of their parent-child sexual communication. They report more frequent communication episodes than their child counterparts [26]. This is problematic because effective parent-child sexual communication has been shown to involve regular dialogue rather than one-off discussions [26,27]. This implies that parents may need prompting to remember to engage in regular open communication on sexual health topics with their children.

Finally, the quality of parent-child sexual communication may be low. This may be because of parental discomfort, low self-efficacy, or limited sexual health knowledge [23,28-31]. If parents are misinformed, they may engage in conversations that are inaccurate or inappropriate. This includes conducting inappropriately gendered parent-child sexual communication, such as providing insufficient information on sociosexual issues [32] or reinforcing archaic or negative gender stereotypes [33]. The risk of this appears to be amplified when parent-child sexual communication involves sexual or gender minority children [34]. In these circumstances, parents have been observed to conduct inappropriately heteronormative parent-child sexual communication or overly prioritize discussions on the risk of HIV and other sexually transmitted infections over other aspects of sexuality education [35].

For these reasons, there are ongoing attempts to support parents to engage in better parent-child sexual communication. This has included in-person workshops [36], web-based learning [37-39], as well as intergenerational activities for parents and children to engage in together such as joint homework [40] and online games [41]. Yet, despite these efforts, parental engagement with parent-child sexual communication educational resources remained low [42]. It is therefore crucial to identify what motivates parents to adopt tools that may improve parent-child sexual communication.

### Technology Acceptance Model

To examine the factors that are associated with parents’ intention to use an app to support parent-child sexual communication, we used a modified version of the technology acceptance model (TAM) [43]. Davis developed the TAM as an extension of Ajzen and Fishbein’s theory of reasoned action (TRA) [44]. The TRA predicts human behavior as determined by different cognitions including attitudes toward the behavior and subjective norms. Attitudes toward the behavior were defined as an individual’s feelings toward performing the behavior, whether negative or positive [44]. The TRA was thus used as a conceptual framework for modeling intention to perform voluntary behaviors. The TRA was then adapted in the form of the TAM to apply theories of voluntary behavior to user acceptance and utilization of computer technologies.

The TAM has been previously used as a conceptual framework to assess parental acceptance of educational digital tools [45]. This study is, however, the first to apply the TAM to understand parental intentions to use technology to improve their parent-child sexual communication. According to the TAM, *intention to use* predicts actual system usage. The attitudes that determine *intention to use* are defined as the technology’s *perceived usefulness* and *perceived ease of use*.

**Figure 1:**
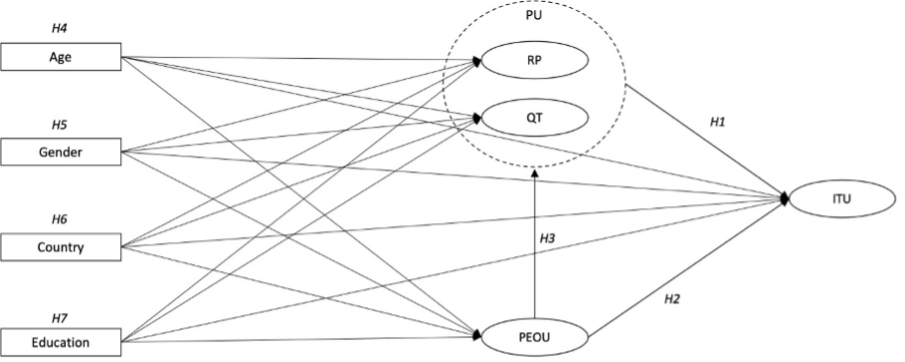
Technology acceptance model (TAM) framework. Rectangles represent observed (measured) variables, and ellipses indicate latent constructs. The dotted circle denotes a second-order latent construct, where *perceived usefulness* is composed of *relevance to parenting* and *quality of technology*. Arrows indicate hypothesized relationships between variables. ITU: intention to use; PEOU: *perceived ease of use*; PU: *perceived usefulness*; QT: *quality of technology*; RP: *relevance to parenting*.

Perceived usefulness reflects the degree to which a person believes the technology will enhance their job performance. According to the TAM, perceived usefulness has a positive association with intention to use. Thus, individuals should be more likely to adopt technologies that they perceive as beneficial and aligned with their goals. In the context of this study, we hypothesize that parents will be more inclined to use the app if they perceive the digital tool as useful in supporting their parent-child sexual communication (Figure 1).

We adapted the TAM to fit our study’s context by inputting elements of the TAM2, in which additional measured factors were added to shape *perceived usefulness* [46]. In our framework, *perceived usefulness* is conceptualized as a second-order latent variable determined by the *quality of technology* and *relevance to parenting*. This decision reflects a theoretical alignment with later iterations of the TAM, in which job relevance (*relevance to parenting*) and output quality (*quality of technology*) are recognized subcomponents of *perceived usefulness* [46]. By embedding *relevance to parenting* and *quality of technology* as dimensions of *perceived usefulness*, our framework acknowledges that parents’ perceptions of usefulness are inherently multidimensional. We do, however, adapt the TAM such that *quality of technology* and *relevance to parenting* do not only influence *perceived usefulness* but instead act as its subcomponents.

Hypothesis: *Perceived usefulness* has a direct and positive association with *intention to use* (H1).

*Perceived ease of use* may be defined as the extent to which a person believes that using the technology will be free of effort. Perceived ease of use is expected to be positively associated with *intention to use*. If a user finds a technology intuitive and accessible, they are anticipated to be more likely to engage with it [47]. Thus, an app that is easy to navigate should be more appealing to parents and encourage usage (Figure 1).

In our study, we examined parents’ *perceived ease of use* of an intergenerational app. We considered that parents might inherently find digital tools difficult to use but may perceive this technology as easier due to its intergenerational design. Specifically, knowing their digitally native children could assist them may positively influence their overall perception of the app’s ease of use [48]. Thus, our assessment of *perceived ease of use* incorporated both parents’ own perceptions and their views regarding their children’s digital fluency.

Hypothesis: *Perceived ease of use* has a direct and positive association with *intention to use* (H2).

*Perceived ease of use* is also hypothesized to be positively associated with perceived usefulness, forming an indirect pathway to intention to use [47]. This hypothesis identifies the interplay between the cognitive effort to use a technology and the perception of its functional benefits. Thus, if parents find an app easier to use, they may be more likely to recognize its utility in enhancing parent-child sexual communication. This may then mean that they are more likely to intend to adopt its usage (Figure 1).

Hypothesis: *Perceived ease of use* has a direct and positive association with *perceived usefulness* (H3).

We hypothesized that parents’ sociodemographic factors would be associated with the latent constructs measured in our model (*relevance to parenting*, *quality of technology*, *perceived ease of use*, and *intention to use*; Figure 1). These factors included age, gender, country of residence, and education level.

We based the relevance of age in our model on previous research identifying the digital divide between adults of different ages [49]. Thus, older generations may feel that they lack the confidence or skills required to effectively engage with an intergenerational app. We anticipated that younger parents may be more accustomed with digital tools and therefore find them easier to use, more useful, and be more willing to engage in technology usage.

We anticipated that parents’ gender would also be significantly associated with our first-order latent constructs. Previous research has identified that fathers engage in parent-child sexual communication less frequently than mothers [50]. When fathers educate their daughters on sexual health, they also discuss fewer topics with a more narrowed focus [29]. We therefore predicted that fathers would be less likely to see the app as relevant to their parenting, thus seeing the app as less useful and having reduced intention to use the digital tool.

Parents’ *perceived usefulness*, *perceived ease of use*, and their *intention to use* may also be influenced by their perceived social norms. Social norms refer to collective beliefs about typical and appropriate beliefs, behaviors, and actions within a group [51]. These norms are internalized during the socialization process and shaped by cultural and educational contexts [52]. Consequently, our study also examined the influence of the parents’ country of residence and education level on the latent constructs. We hypothesized that parents residing in more liberal countries within Europe would be more inclined to engage with parent-child sexual communication digital technologies, whereas parents from more conservative regions would be less willing. Furthermore, consistent with prior research on in-person school-based sexuality education [53], we anticipated that higher parental educational attainment would be associated with greater parental support of sexuality education and thus a higher intention to use a parent-child sexual communication app.

Hypotheses: Age (H4), gender (H5), country of residence (H6), and education level (H7) may be associated with *relevance to parenting*, *quality of technology*, *perceived ease of use*, and *intention to use*.

## Methods

### Recruitment

This work represents the second phase of a multifaceted survey on parental attitudes toward digital sexuality education [54]. In the first phase, we examined parental views on the age at which adolescents should receive sexuality education topics via a digital app. The present study builds on this by focusing not on the age-appropriateness of content but on parents’ intention to use multigenerational digital tools to improve parent-child sexual communication. Data collection occurred between May and June 2022 across 3 regions: Belgium (the Flemish area), the United Kingdom, and Italy. These locations were selected to capture a diverse spectrum of perspectives on sexual and gender diversity within Europe, as reflected by ILGA-Europe rankings. ILGA-Europe assesses the legal and policy landscape for gender and sexual minority people across European countries. It calculates an index score based on the country’s laws on equality, gender recognition, family rights, and hate crimes, as well as their tolerance toward freedom of expression. In the 2022 survey, Belgium ranked highly, the United Kingdom received an intermediate ranking, and Italy scored relatively poorly for their lack of inclusive laws and policies [55].

Data were gathered through an anonymous online survey administered by the official polling agency iVOX [56]. iVOX is a Belgian company that holds the largest consumer panel in Belgium. It has been incorporated by the Bilendi research marketing agency, which enables access to panels across European countries. A total of 1500 individuals were recruited from iVOX’s closed survey online panels, which comprise between 30,000 and 250,000 members per region. Members voluntarily participated in the surveys and were compensated with gift vouchers upon completion. Members were provided with an informed consent form to complete prior to enrollment. Data were pseudoanonymized at the point of collection.

Eligible participants were adult parents of children aged 9-18 years. The lower age threshold of 9 years was chosen to reflect the age at which many children start to engage with more complex sexuality education in schools [1]. Inclusion criteria required participants to provide essential demographic details such as ethnicity, gender, and sexual identity, as well as complete key sections of the survey (Multimedia Appendix 1). Participants were excluded if they failed to meet these criteria or if their engagement with the survey was deemed insufficient. Insufficient engagement was determined by low or absent variability in response to Likert scale questions, which was presumed to indicate poor attention to the survey content.

### Survey Instrument

The survey was administered in English, Dutch, and Italian. The survey was developed in English, and back-translation was performed by native Dutch and Italian speakers (JT and LC, respectively) to ensure linguistic consistency. Before completing the survey, participants were briefed on the hypothetical app. The app was described as a complement to school sexuality education, which would release material in accordance with the school curriculum. It would be available in parent and child versions to download onto their smartphones. To minimize response errors, the questionnaire included working definitions of sexuality education and parent-child sexual communication [57].

Participants reported on individual and family characteristics. Individual characteristics included age, which was nonparametric and presented as median (IQR). Categorical individual characteristics included gender, ethnicity, education level, sexuality, relationship status, and religion. Religiosity was evaluated on a 5-point Likert scale (“I am a religious person”; 1=strongly disagree to 5=strongly agree) and reported as mean (SD). Sociosexual attitude was assessed using items from the Sociosexual Orientation Inventory [58]. Participants rated 3 statements on a 5-point Likert scale (1=strongly disagree to 5=strongly agree): “sex without love is okay,” “I can imagine myself being comfortable and enjoying ‘casual’ sex with different partners,” and “I do not want to have sex with a person unless I am sure that we will have a long-term serious relationship” (reverse coded). Sociosexual attitude was expressed as a composite mean (SD).

Family characteristics included the number of children in the family and the age of the oldest child. These items were nonparametric and expressed as median (IQR). Parents also provided their perceptions of their children’s gender and sexual identities. As their children were not surveyed in parallel, we could not confirm the veracity of the perceived identities.

The latent constructs *intention to use*, *perceived ease of use*, *quality of technology*, and *relevance to parenting* were measured using a minimum of two items each (Table 1) [59]. The items for *relevance to parenting* were directly obtained from Pariera [23]. In contrast, the measured items for the other constructs were not previously validated, and their development was informed by prior research instead [23,38,41,60]. To ensure content validity, a panel of 7 European experts in sexuality education and child development reviewed all items. The panel recommended minor adjustments, which were enacted prior to study commencement. All items were rated on a 5-point Likert scale (1=strongly disagree to 5=strongly agree).

**Table 1:** First-order latent constructs.

Item			Item question
**Intention to use (ITU)**			
	ITU1	I would like my child or children to use the app.	
	ITU2	I would like to use the app to aid communication with my child about sexual health.	
	ITU3	I think that the app would improve my child’s sexuality education.	
**Perceived ease of use (PEOU)**			
	PEOU1	I find apps easy to use.	
	PEOU2	My child or children find apps easy to use.	
**Quality of technology (QT)**			
	QT1	As a parent, I should be able to have my own profile on the app.	
	QT2	The app should include information sections for me to learn from.	
	QT3	The app should include example scripts on difficult topics for me to use when talking to my child about sexual health.	
	QT4	The app should include mock scenarios that I can discuss with my child.	
	QT5	The app should include educational games for me to play with my child.	
	QT6	The app should allow my child to flag topics to me that they would like to discuss in person.	
	QT7	The app should be able to send me alerts to encourage me to broach important or relevant topics with my child.	
**Relevance to parenting (RP)**			
	RP1	I think it is important for parents to talk to their children about sex.	
	RP2	I think parent-child communication about sex has a positive effect on young people.	

### Statistical Analysis

Demographic data were presented as frequencies for the total sample and stratified by region.

Exploratory factor analysis (EFA) was conducted to identify the dimensionality of the survey instrument. Analysis was performed using maximum likelihood extraction with oblique rotation (oblimin). Confirmatory factor analysis (CFA) was then performed using maximum likelihood with robust standard errors. This was because items were not normally distributed, as determined by the Andersen-Darling tests (*P*<.001) [61]. Internal consistency was evaluated using composite reliability. Convergent validity was assessed using average variance extracted (AVE), and discriminant validity was evaluated with the Fornell-Larcker criterion [62]. Harman’s single-factor test was used to assess for common method bias [63]. Structural equation modeling (SEM) was then applied to test the study hypotheses. Model fits were assessed using multiple commonly used indices—comparative fit index (CFI), Tucker-Lewis Index (TLI), root-mean-square of residuals (RMSR), and root-mean-square error of approximation (RMSEA)—to provide a robust and well-rounded evaluation of fit [64].

Initially, *perceived usefulness* was conceptualized as a latent variable with its own measurement items. However, preliminary analysis revealed that the items developed for *perceived usefulness* did not consistently load onto a factor. To address this, *perceived usefulness* was respecified as a second-order latent variable determined by *quality of technology* and *relevance to parenting*. Such higher-order modeling within SEM has been previously performed in other contexts [65]. Thus, in our model, parents’ perceived usefulness of a hypothetical parent-child sexual communication app was composed of the quality of the technology and how relevant they deemed the app to their parenting. This adjustment deviates from the TAM but was deemed theoretically plausible and empirically necessary to ensure good alignment between measurement items and their latent constructs. A CFA was performed to assess the loading of *quality of technology* and *relevance to parenting* on the second-order construct *perceived usefulness*. This identified good CFI (0.95) and TLI (0.93), and acceptable RMSEA (0.08). Thus, the adjustment from the original model was empirically justified. Our approach also aligns with prior extensions of TAM in educational contexts, where the original variables of the TAM have been modeled as higher-order constructs to capture multiple elements [45]. Indeed, a recent meta-analysis on the modern use of TAM emphasized the importance of improving the predictive power of technology adoption models by tailoring the TAM to the specific context of the study [47].

The observed constructs—age, gender, country of residence, and education level—were added to the SEM in accordance with our TAM framework. Age was a scale variable. Gender, country of residence, and education level were categorical and therefore inputted with dummy variables. The reference groups for the categorical variables included in the SEM were female, the United Kingdom, and primary education level.

All analyses considered a 2-tailed *P* value of <.05 as statistically significant. This study has been reported in adherence with the Checklist for Reporting Results of Internet e-Surveys (CHERRIES) [66].

### Ethical Considerations

Ethics approval was granted on May 25, 2022, by the KU Leuven Social and Societal Ethics Committee (SMEC reference: G-2022-5160). Informed consent was obtained from all participants before survey engagement. Data were anonymized and required and stored securely to ensure confidentiality and compliance with ethical standards. Participants were compensated with gift vouchers through the online platform.

## Results

### Sample Characteristics

Demographic data are summarized in Table 2. A total of 1296 participants were included in the analysis. The median age of the participants was 46 (IQR 40-51) years. Most participants reached either college-level education (aged 16-18 years) or tertiary education (aged >18 years). The frequencies of gender, ethnic, religious, and sexual minority groups in the sample were low. Only 3.2% (n=42) of study participants identified as noncisgender, 3.9% (n=51) as not heterosexual, and 4.7% (n=61) considered themselves of non-European ethnicity. Similarly, most individuals (n=785, 60.6%) considered themselves Christian. There were, however, a sizeable minority of individuals who identified as not having a religion (n=459, 35.4%). This likely impacted religiosity, where most participants across the 3 regions gave intermediate scores (Table 2). Nearly 89% (n=1150) of participants considered themselves to be in a committed relationship, and the sociosexual attitude scores were intermediate (composite mean 2.89 on a Likert scale from 1 to 5; Table 2). With respect to family characteristics, the median number of children per family was 2 (IQR 1-2). This approximates the total fertility rate across countries in the European Union [67]. The median age of the eldest child of parents surveyed in our sample was 15 (IQR 12-17) years. Across all regions, fewer than 10% of parents perceived their children to identify as a minority gender or sexuality group (n=30 [2.3%] and n=67 [5.2%], respectively).

**Table 2:** Demographic characteristics.

Demographic characteristics		Total (N=1296)		Region				
				Belgium (n=463)		United Kingdom (n=426)		Italy (n=407)
**Parent characteristics** **, median (IQR)**								
	Age^a^ (years)	46 (40-51)	45 (41-50)		45 (39-50)		45 (40-51)	
**Gender, n (%)**								
	Cisgender female	653 (50.4)	232 (50.1)		236 (55.4)		185 (45.5)	
	Cisgender male	601 (46.4)	231 (49.9)		176 (41.3)		194 (47.7)	
	Any other gender identity	42 (3.2)	0 (0)		14 (3.3)		28 (6.9)	
**Ethnicity, n (%)**								
	European	1235 (95.3)	457 (98.7)		376 (88.3)		402 (98.8)	
	Any other ethnicity	61 (4.7)	6 (1.3)		50 (11.7)		5 (1.2)	
**Education level, n (%)**								
	Primary school (≤12 years)	18 (1.4)	2 (0.4)		2 (0.5)		14 (3.4)	
	Secondary education (12-16 years)	165 (12.7)	15 (3.2)		73 (17.1)		77 (18.9)	
	College education (16-18 years)	529 (40.8)	162 (35.0)		144 (33.8)		223 (54.8)	
	Tertiary education (>18 years) and above	584 (45.1)	284 (61.3)		207 (48.6)		284 (22.9)	
**Religion, n (%)**								
	Christianity	785 (60.6)	260 (56.2)		204 (47.9)		321 (78.9)	
	Any other religion	52 (4.0)	9 (0.7)		27 (6.3)		16 (3.9)	
	No religion	459 (35.4)	194 (41.9)		195 (45.8)		70 (17.2)	
Religiosity^a^, mean (SD)		2.6 (1.3)		2.2 (1.1)		2.3 (1.3)		3.2 (1.2)
**Sexuality, n (%)**								
	Heterosexual	1245 (96.1)	438 (94.6)		413 (96.9)		394 (96.8)	
	Any other sexuality	51 (3.9)	25 (5.4)		13 (3.1)		13 (3.2)	
**Relationship status, n (%)**								
	In a committed relationship	1150 (88.7)	411 (88.8)		364 (85.4)		375 (92.1)	
	In a casual relationship	28 (2.2)	8 (1.7)		11 (2.6)		9 (2.2)	
	Single	118 (9.1)	44 (9.5)		51 (12.0)		23 (5.7)	
Sociosexual attitude^b^, mean aggregate value (SD)		2.89 (1.0)		2.9 (1.0)		3.0 (1.0)		2.8 (1.0)
**Family characteristics** **, median (IQR)**								
	Number of children	2 (1-2)	2 (1-3)		2 (1-3)		2 (1-2)	
	Age of the oldest child (years)	15 (12-17)	15 (12-17)		15 (12-17)		14 (11-16)	
**Parental perception of their children’s gender, n (%)**								
	Cisgender female	315 (24.3)	110 (23.8)		106 (24.9)		99 (24.3)	
	Cisgender male	387 (29.9)	122 (26.3)		114 (26.8)		151 (37.1)	
	Cisgender female and male	540 (41.7)	213 (46.0)		187 (43.9)		140 (34.4)	
	One or more transgender, gender fluid, or nonbinary child	30 (2.3)	7 (1.5)		15 (3.5)		8 (2.0)	
	Their parent does not know	24 (1.0)	11 (2.4)		4 (0.9)		9 (2.2)	
**Parental perception of their children’s sexuality, n (%)**								
	Heterosexual	1036 (79.9)	336 (72.6)		331 (77.7)		369 (90.7)	
	One or more homosexual, bisexual, or any other sexuality child	67 (5.2)	30 (6.5)		31 (7.3)		6 (1.5)	
	Their parent does not know	193 (14.9)	97 (21.0)		64 (15.0)		32 (7.9)	

^a^Data were measured on a 5-point Likert scale (“I am a religious person”; 1=strongly disagree to 5=strongly agree).

^b^Data were measured on a 5-point Likert scale reflecting (1=strongly disagree to 5=strongly agree) increasing sexual liberalism.

### Measurement Model

The item-level correlation matrix alongside the items’ means (SD) is presented in Multimedia Appendix 2. EFA identified 4 factors with eigenvalues >0.5 (Table 3). These factors were assigned based on item loadings ≥0.4, consistent with the recommendations of Stevens [68]. This threshold ensured robust item-factor associations, with all items aligning well with the model’s theorized latent constructs. Sample adequacy was confirmed by a Kaiser-Meyer-Olkin statistic of 0.83 [69]. This provided a strong basis for factor analysis. The EFA model demonstrated excellent fit indices: TLI=0.95, RMSEA=0.06, and RMSR=0.02 [70]. The highest interfactor correlation was 0.54, which was below the recommended upper threshold of 0.7 [71]. This supported our assumption of factor independence.

**Table 3:** Exploratory factor analysis.

	Intention to use (ITU)	Perceived ease of use (PEOU)	Quality of technology (QT)	Relevance to parenting (RP)
ITU1	0.86	—^a^	—	—
ITU2	0.75	—	—	—
ITU3	0.79	—	—	—
PEOU1	—	0.88	—	—
PEOU2	—	0.95	—	—
QT1	—	—	0.57	—
QT2	—	—	0.75	—
QT3	—	—	0.81	—
QT4	—	—	0.73	—
QT5	—	—	0.48	—
QT6	—	—	0.64	—
QT7	—	—	0.63	—
RP1	—	—	—	0.78
RP2	—	—	—	0.90

^a^Not applicable.

To validate the factor structure identified in the EFA, CFA was used (Table 4). The 4-factor CFA model demonstrated similarly excellent fit indices: CFI=0.96, TLI=0.95, and RMSEA=0.06. These values met or exceeded benchmark standards for model fit (CFI>0.9, TLI>0.9, and RMSEA<0.08) [70]. All standardized factor loadings were greater than 0.4, further reinforcing the validity of the associations between items and their respective factors (Multimedia Appendix 3) [68]. Measurement reliability analyses using composite reliability provided additional support as they identified excellent internal consistency (Table 4).

**Table 4:** Confirmatory factor analysis for first-order latent constructs.

Items	Composite reliability	Average variance extracted
Intention to use	0.86	0.67
Perceived ease of use	0.91	0.84
Quality of technology	0.86	0.48
Relevance to parenting	0.83	0.71

Convergent validity was evaluated using AVE. Three constructs (*intention to use*, *perceived ease of use*, and *relevance to parenting*) achieved AVE values above the recommended threshold of 0.5, reflecting strong convergent validity (Table 4). While the AVE for the *quality of technology* construct was slightly below this threshold at 0.48, it was deemed acceptable given its proximity to the cutoff and the exploratory nature of the study. This was considered appropriate given the overall robustness of the CFA results. Discriminant validity was confirmed using the Fornell-Larcker criterion. Furthermore, Harman’s single-factor test revealed that less than 50% of the variance was explained by a single construct model (32%). This alleviated concerns regarding common method bias. Together, these findings confirmed the appropriateness of the model for capturing the intended constructs.

### Structural Model

The SEM is presented in Table 5. The SEM demonstrated good fit indices (CFI=0.94, TLI=0.93, and RMSEA=0.05), demonstrating that the model adequately represented the data. The explanatory power of the model was *R*^2^=0.47.

*Perceived usefulness* emerged as a dominant determinant of intention to use, demonstrating a strong and statistically significant direct association (β=0.67, *P*<.001). This supports hypothesis H1. However, perceived ease of use did not show a significant relationship with either intention to use (β=–0.01, *P*>.05) or perceived usefulness (β=0.10, *P*>.05). These findings suggested that perceived ease of use, which we had anticipated was a key component of our TAM, did not influence participants’ perception of usefulness or their intention to use the parent-child sexual communication app. We therefore rejected hypotheses H2 and H3.

Age was significantly negatively associated with *intention to use* (β=–0.06, *P*=.05). This implied that younger parents were significantly more likely to intend to use the tool to assist with their parent-child sexual communication. Yet surprisingly, this was not explained by any significant relationship between age and the other latent constructs. This included *perceived ease of use*.

Our model identified that male gender had a significant negative association with *relevance to parenting* (β=–0.15, *P*<.001). This indicated that male parents considered the tool less relevant for parent-child sexual communication than their female counterparts. Conversely, male gender was positively associated with *intention to use* (β=0.08, *P*=.01). This suggested that despite its decreased relevance to parenting, men expressed a higher intention to use the digital tool overall. Participants of gender minority groups similarly displayed a significant positive association with *intention to use* (β=0.06, *P*=.05) when compared with their female counterparts. They however had a significant negative association with *perceived ease of use* (β=–0.09, *P*=.05). Thus, compared to female parents, they were less likely to perceive apps as easy to use. However, due to the small sample size of gender minority parents (42/1296, 3.2%), these results should be interpreted cautiously and considered exploratory.

Parents’ country of residence also demonstrated significant associations with several constructs. Compared with respondents from the United Kingdom, Belgians demonstrated significant negative associations with *quality of technology* (β=–0.12, *P*=.001), *perceived ease of use* (β=–0.07, *P*=.004), and *intention to use* (β=–0.14, *P*<.001). Thus, the only construct that was not significantly associated with was *relevance to parenting*. This suggests significant cultural differences in technology acceptance between the United Kingdom and Belgium. Conversely, parents from Italy voted similarly to parents from the United Kingdom, except they were significantly less likely to perceive the technology as easy to use (β=–0.79, *P*<.001).

College-educated participants exhibited significant positive associations with *relevance to parenting* (β=0.25, *P*=.05), suggesting increased perceived relevance for parenting among more educated parents. Similarly, those with tertiary or higher education also showed significant positive associations with *relevance to parenting* (β=0.27, *P*=.04). No significant relationships emerged between secondary education level and any of the latent constructs.

**Figure 2:**
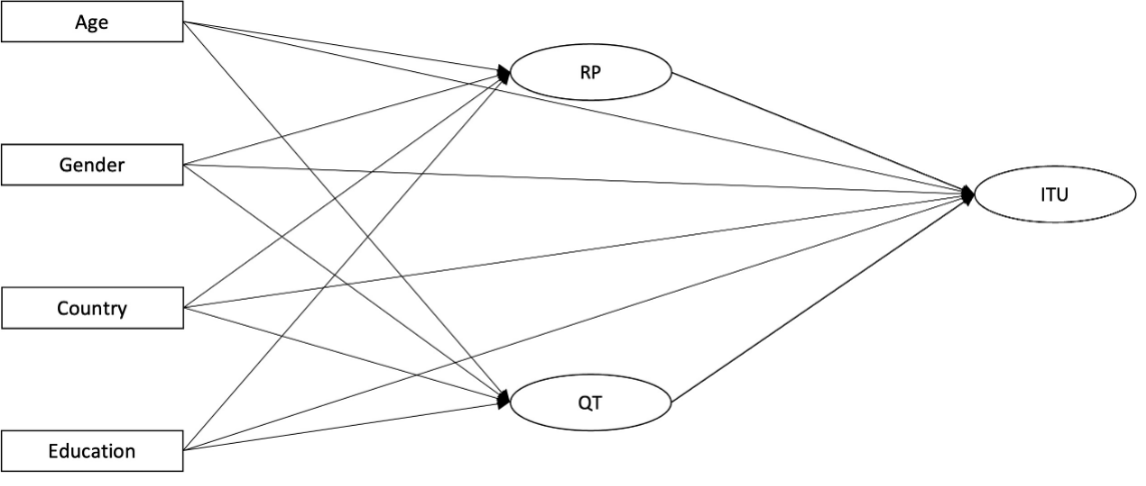
Alternative 3-latent-construct model. Rectangles represent observed (measured) variables, and ellipses indicate latent constructs. Arrows indicate hypothesized relationships between variables. ITU: intention to use; QT: quality of technology; RP: relevance to parenting.

Given that *perceived usefulness* was the only observed significant latent construct associated with *intention to use*, we sought to further understand the relative contributions of its subcomponents—*relevance to parenting* and *quality of technology*. A secondary analysis was therefore conducted by constructing an alternative 3-latent-construct model (Figure 2).

This 3-latent-construct model retained good fit indices (CFI=0.91, TLI=0.89, and RMSEA=0.06). The explanatory power of the model was, however, reduced to *R*^2^=0.38. Within this model, both *relevance to parenting* and *quality of technology* had a significant positive association with *intention to use* (β=0.09, *P*=.002 and β=0.59, *P*<.001, respectively). The altered estimates and *P* values for the measured variables are presented in Multimedia Appendix 4 and show equivalent results to the full TAM framework.

**Table 5:** Structural equation modeling. *P* values are only listed when significant at <.05.

Construct		Unstandardized β	Standardized β	*P* value
**H1** **:** ***Perceived usefulness*** **(PU) has a direct and positive association with** ***intention to use*** **(ITU)**				
	PU → ITU	1.36	0.67	<.001
**H2:** ***Perceived ease of use*** **(PEOU) has a direct and positive association with** ***intention to use*** **(ITU)**				
	PEOU → ITU	–0.00	–0.01	—
**H3:** ***Perceived ease of use*** **(PEOU) has a direct and positive association with** ***perceived usefulness*** **(PU)**				
	PEOU → PU	0.04	0.10	—
**H4: Age has a direct association with** ***quality of technology*** **(QT),** ***relevance to parenting*** **(RP),** ***perceived ease of use*** **(PEOU), and** ***intention to use*** **(ITU)**				
	Age → QT	0.00	–0.01	—
	Age → RP	0.00	0.03	—
	Age → PEOU	0.00	0.00	—
	Age → ITU	–0.01	–0.06	.05
**H5: Gender has a direct association with** ***quality of technology*** **(QT),** ***relevance to parenting*** **(RP),** ***perceived ease of use*** **(PEOU), and** ***intention to use*** **(ITU)^a^**				
	Male gender → QT	–0.01	–0.01	—
	Male gender → RP	–0.20	–0.15	<.001
	Male gender → PEOU	0.04	0.02	—
	Male gender → ITU	0.11	0.08	.01
	Other gender identity → QT	0.10	0.05	—
	Other gender identity → RP	–0.21	–0.06	—
	Other gender identity → PEOU	–0.51	–0.09	<.001
	Other gender identity → ITU	0.24	0.06	.05
**H6: Country of residence has a direct association with** ***quality of technology*** **(QT),** ***relevance to parenting*** **(RP),** ***perceived ease of use*** **(PEOU), and** ***intention to use*** **(ITU)^b^**				
	Belgium → QT	–0.10	–0.12	.001
	Belgium → RP	0.04	0.03	—
	Belgium → PEOU	–0.14	–0.07	.004
	Belgium → ITU	–0.21	–0.14	<.001
	Italy → QT	0.03	0.04	—
	Italy → RP	0.08	0.06	—
	Italy → PEOU	–1.77	–0.79	<.001
	Italy → ITU	0.02	0.02	—
**H7: Education level has a direct association with** ***quality of technology*** **(QT),** ***relevance to parenting*** **(RP),** ***perceived ease of use*** **(PEOU), and** ***intention to use*** **(ITU)^c^**				
	Secondary education → QT	0.13	0.12	—
	Secondary education → RP	0.20	0.10	—
	Secondary education → PEOU	0.18	0.06	—
	Secondary education → ITU	0.10	0.05	—
	College education → QT	0.16	0.20	—
	College education → RP	0.33	0.25	.05
	College education → PEOU	0.16	0.08	—
	College education → ITU	0.19	0.13	—
	Tertiary or higher education → QT	0.12	0.15	—
	Tertiary or higher education → RP	0.35	0.27	.04
	Tertiary or higher education → PEOU	0.19	0.09	—
	Tertiary or higher education → ITU	0.17	0.12	—

^a^Reference groups are female.

^b^Reference groups are the United Kingdom.

^c^Reference groups are primary education level.

## Discussion

### Principal Findings and Comparison With Previous Works

This work examined the potential for a hypothetical intergenerational app designed to enhance parent-child sexual communication and align parental efforts with school-based sexuality education. Using a modified TAM [43], we explored factors that may relate to parents’ intention to use the proposed digital tool. We sampled across 3 distinct regions in an attempt to obtain perspectives of parents across the spectrum of European opinion. Belgium, the United Kingdom, and Italy were selected because they differ in sociopolitical contexts: Belgium has progressive sexuality education policies, the United Kingdom is in an intermediate position, and Italy lacks a national mandate for sexuality education and holds more conservative social norms [55]. These contextual contrasts were mirrored in our descriptive statistics: Italian parents reported higher religiosity and fewer nonheterosexual identities, while parents from Belgium and the United Kingdom were more open to their children having alternative gender and sexual identities. Together, these findings suggest that both sociopolitical climate and demographic composition shaped parents’ responses to the hypothetical technology.

We identified that *perceived usefulness* is significantly associated with participants’ *intention to use* the app. This aligns with one of the foundational premises of the TAM, which posits that *perceived usefulness*—the degree to which an individual believes that using the technology will enhance their performance—is a critical driver of technology adoption [43]. The importance of *perceived usefulness* has been observed in previous studies on user adoption of new educational information technologies, including multimedia learning environments [72,73]. The dominance of *perceived usefulness* underscores that, in our context, parents’ intentions are primarily related to their perception of the app’s utility in addressing their parent-child sexual communication needs.

To further investigate the relative contributions of the first-order constructs, which comprised *perceived usefulness*, *relevance to parenting*, and *quality of technology*, we proposed an alternative 3-latent-construct model. While both models demonstrated good fit indices, the TAM-based model explained a greater proportion of variance in *intention to use* (*R*^2^=0.47) compared to the 3-latent-construct model (*R*^2^=0.38). In the 3-latent-construct model, both *relevance to parenting* and *quality of technology* significantly influenced *intention to use*. These findings align with prior studies on intergenerational sexuality education tools. Parents who ascribe value to sexuality education were more likely to be motivated to engage in new technologies to improve their parent-child sexual communication [42]. Furthermore, we identified the critical role of reliable, engaging, and content-rich technologies in facilitating parental adoption of digital tools [41]. In this work, features deemed attractive by parents included example scripts, interactive games, mock scenarios, and alert features. Similarly, surveys of children and adolescents have emphasized the importance of high-quality app features in driving user adoption of sexuality education technologies [60,74].

Alongside identifying the significant positive association of *perceived usefulness* with *intention to use*, we also identified that sociodemographic factors were significantly associated with parents’ intention to use the digital technology. Younger parents were more likely to intend to use the sexuality education app, which is in keeping with their comfort with digital educational adjuncts [75]. Furthermore, parents who did not identify as female were also more likely to intend to use the app. This may be because mothers feel more self-efficacy and confidence engaging in parent-child sexual communication and therefore do not see the same utility [18]. Notably, the country of residence was also an important determinant of survey responses. Belgian parents reported lower perceived ease of use, technology quality, and intention to use compared with British parents, despite broadly similar demographic profiles. This may reflect differences in cultural expectations around school-parent collaboration, with Belgium’s comprehensive sexuality education perhaps reducing the perceived need for supplementary tools [76]. Conversely, in Italy, lower ease of use ratings may be linked to the more limited digital literacy when compared to its European neighbors [77]. These differences suggest parents across Europe will interact differently with virtual sexuality education technologies, and that national policies, sociocultural norms, and digital confidence must be considered when adapting such tools. Finally, we identified that the relative importance ascribed to parent-child sexual communication increased among parents who had higher educational attainment. This has been previously identified in other settings [78,79].

Unlike *perceived usefulness* or sociodemographic factors, *perceived ease of use* was not significantly associated with the other constructs in the model. This diverged from the classical TAM and contrasts with prior research on education technologies, which found that poor usability negatively affected parental engagement in their children’s digital education [45]. In those cases, parents were less engaged in their child’s online learning when they perceived the technology as difficult to use. However, our findings suggest that parents’ intention to use an intergenerational app to support parent-child sexual communication was independent of its usability. Instead, parents were willing to engage with educational technology they found useful, regardless of how easy it was for them or their children to navigate. This discrepancy may be because our study only examined parents’ theoretical intention to use, rather than their actual usage behaviors. Alternatively, it may reflect contextual differences, as the referenced study examined parents’ technology usage to facilitate children’s home-based learning during the global COVID-19 pandemic [45]. Given the heightened parental stress during this period, which negatively impacted children’s learning outcomes [80], parents may have had reduced emotional and cognitive capacities to engage effectively with new or challenging educational technologies.

Our work is also not the first study to observe a diminished role for *perceived ease of use* within the TAM. Several studies on online educational platforms have similarly found that *perceived ease of use* does not significantly predict *intention to use* [81,82]. However, unlike our study, these works primarily sampled Generation Z students (born between 1995 and 2012) [83]. As “digital natives” who grew up with internet and mobile phone technologies [84], Generation Z may be less concerned with ease of use due to their familiarity and adaptability with new digital tools. In contrast, our study population consisted of parents with a minimum survey age of 18 years and a median age of 46 (IQR 40-51) years. It may therefore be that parents are willing to overlook usability challenges if they perceive the technology as highly useful or important, such as in the context of parent-child sexual communication. Yet, we also observed that lower parental ages were associated with greater intention to use the technology. This implies that younger parents may have the additional benefit of feeling comfortable tackling new technology challenges, independent of their current perceived ease of use.

### Limitations

There are several limitations to the study that warrant discussion. First, the study relied on an online polling agency and a closed survey panel for recruitment, which may have introduced sampling bias. This may have introduced a self-selection bias, where only individuals motivated and interested in the topic with sufficient free time took part in the survey. To reduce the risk of this and increase overall motivation to engage with the project, we ensured that participants were adequately compensated for their involvement. Yet, there remained a predominance of cisgender, heterosexual, Christian, and European ethnicity participants in the sample. This limited diversity constrains the extent to which our findings can be applied to broader and more diverse parent populations. However, this demographic composition aligns with region-specific census data in the United Kingdom, which is the only region studied here with detailed census records available [85]. Nevertheless, it would be important to survey parents and children from backgrounds underrepresented here, who may have different attitudes toward digital parent-child sexual communication tools [13,34]. Future work should therefore use inclusive recruitment strategies to capture the parental attitudes among minority groups, such as community partnerships and targeted oversampling of underrepresented groups. Qualitative approaches such as focus groups or in-depth interviews may also provide richer insights from parents underrepresented in online survey panels.

Second, the hypothetical nature of the app may have constrained participants’ engagement with the survey items. Unlike studies evaluating real-world interventions [42], our work relied on participants’ imagination to envision the app’s features, utility, and their intention to use it. This reliance on the hypothetical may have reduced the real-world applicability of our findings, as stated intention may not translate to actual adoption and engagement in the technology. There is a potential gap between an individual’s intent to use and actual usage behaviors, as has been observed among consumers in other settings [86]. Furthermore, causality could not be established due to the cross-sectional nature of the study. While our model provides insights into relationships between constructs, future longitudinal or experimental studies with app prototypes are necessary to determine causal effects and understand how parental engagement evolves over time in real-world settings. This evaluation may also include objective usage data, for example, in-app analytics.

Third, there are limitations related to our construction of the specific TAM model. Our *perceived ease of use* construct was also measured using only 2 items (capturing parents’ ease and their perception of their children’s ease). This was to avoid an excessively long and tiring survey, which may have reduced respondent attention and answer quality. Yet while reliability was high, the reduced breadth of our measure may have limited its sensitivity and contributed to the lack of significant associations observed. Future work may therefore expand on our measurement to assess whether these influence parental intent. Furthermore, we modified *perceived usefulness* to a second-order latent construct comprising *quality of technology* and *relevance to parenting*. While this approach was empirically justified, it deviated from the traditional TAM framework [43]. This modification prevented us from evaluating *perceived usefulness* as an original construct. This may limit the comparability of our findings with studies that operationalized *perceived usefulness* as a single first-order construct. However, our decision reflects known extensions of TAM and was therefore both justified. Our decision was in keeping with previous research in educational and e-learning settings, which have adjusted the TAM and its constructs to specific contexts [46]. Furthermore, as the purpose of the TAM is to provide a theoretical springboard for understanding the psychology of technology acceptance, it is necessary to tailor the foundational model to the specific context in which the research is performed. If this is not performed, the predictive capability of the TAM may be limited [47].

### Implications of Study Findings

This study provides insights for researchers, educators, and app developers aiming to enhance parent-child sexual communication through digital interventions.

From a theoretical perspective, our findings add to a growing body of evidence that suggests that *perceived ease of use*—a previously core tenet of the TAM—plays a diminishing role in technology adoptions as digital literacy becomes ubiquitous among younger generations [87]. This shift calls for a re-evaluation of traditional TAM constructs when applied to “digital native” groups who feel comfortable with trialing new technologies. Future studies should investigate whether this trend persists across different cultural and technological settings.

From a practical standpoint, the overarching importance of *perceived usefulness* to parents’ intention to engage in parent-child sexual communication apps should be central to any future app development. We identified that parents who perceive sexuality education as relevant and important were more likely to be motivated to use an app to assist and guide their parent-child sexual communication. Future work should consider how to encourage and inspire less motivated parents to engage in parent-child sexual communication. Our findings directly inform this, as they highlight which sociodemographic groups may be less likely to adopt the technology and why. Tailored information campaigns may then be developed to build trust and highlight the apps’ utility for these groups, as well as explain the benefits of comprehensive sexuality education. For example, our identification of education level as significantly associated with perceived relevance to parenting should be useful to future researchers in this area. Care should be taken to engage parents with lower levels of educational attainment to emphasize the importance of parent-child sexual communication on their children’s development. It is, however, noteworthy that this reduction in perceived relevance to parenting did not affect parents’ overall intention to use the technology.

Our work also underscores the importance of prioritizing high-quality features in app development to improve parent-child sexual communication. The parents surveyed in this study prioritized the quality of technology as a critical determinant of intention to use. This encompassed app content reliability, functionality, multimedia options, and user engagement features. Educators should therefore focus on developing apps that are functional and high specification. However, balancing these desirable features against the associated development and operational costs of such complex app functionalities is essential [88]. Cost-benefit analyses may therefore be helpful to identify the most impactful features to prioritize in resource-constrained settings [89]. Collaboration between developers, schools, and policymakers could also help distribute costs to ensure high-quality tools are equitably accessible to families. Engaging parents through qualitative work in the design process may yield further insights into their preferences and priorities, ensuring that the most desirable app features are incorporated. Additionally, the recent advent of large language models and artificial intelligence technologies offers promising avenues to enhance content customization to the learning needs of the parent-child dyad with the aim to maximize user interaction [90]. Future work may also explore the role of cost-sharing models, such as government subsidies or collaborations between schools, to make development of high-quality parent-child sexual communication tools feasible and the apps freely accessible to families. When a suitable app prototype has been developed, longitudinal studies will be needed to assess how parental engagement evolves as they interact with a parent-child sexual communication app over time. Understanding these dynamics could inform strategies to sustain long-term adoption and maximize the app’s effectiveness. For example, periodic app updates based on user feedback may improve satisfaction and retention [91].

Finally, children’s engagement is integral to the success of any intergenerational interventions [26]. It is therefore imperative to include children in future evaluations of parent-child sexual communication apps. The maturity of the educational content they receive on the app and discuss with their parents [1], as well as their preferences for different app features [92], will evolve as they develop. Sampling across diverse age groups will be helpful to ensure apps resonate with children’s evolving needs and preferences.

### Conclusions

This study contributes novel insights into digital sexuality education technologies aiming to enhance parent-child sexual communication. We identified that *relevance to parenting* and *quality of technology* were the critical determinants of parental intention to use hypothetical sexuality education apps. Our findings contribute to the theoretical advancement of the TAM in the context of parent-child sexual communication and provide actionable recommendations for app developers, researchers, and educators. Future research should involve real-world evaluations of intergenerational sexuality education apps to further refine our understanding and improve communication tools available for parents and their children. This is with the overall aim of ensuring parents can engage in high-quality, unprejudicial, and open communication with their children on sexual health topics.

## Appendix

Multimedia Appendix 1 Inclusion criteria.

Multimedia Appendix 2 Item-level correlation matrix presented with item mean (SD). ITU: intention to use; PEOU: perceived ease of use; QT: quality of technology; RP: relevance to parenting.

Multimedia Appendix 3 Standardized factor loading.

Multimedia Appendix 4 Structural equation modeling for the alternative three latent construct model.

## Abbreviations

AVE: average variance extracted
CFA: confirmatory factor analysis
CFI: comparative fit index
CHERRIES: Checklist for Reporting Results of Internet e-Surveys
EFA: exploratory factor analysis
RMSEA: root-mean-square error of approximation
RMSR: root-mean-square of residuals
SEM: structural equation modeling
TAM: technology acceptance model
TLI: Tucker-Lewis Index
TRA: theory of reasoned action

## Data Availability

Anonymized participant data are available upon reasonable request from the corresponding author.
