# Supplementary figures and images for: Exploring Parental Intentions to Use Digital Tools to Enhance Parent-Child Sexual Communication in Europe: Cross-Sectional Questionnaire Study
Source: JMIR Pediatr Parent. 2025 Oct 10;8:e75489. doi: 10.2196/75489 (PMC12552825; doi:10.2196/75489)

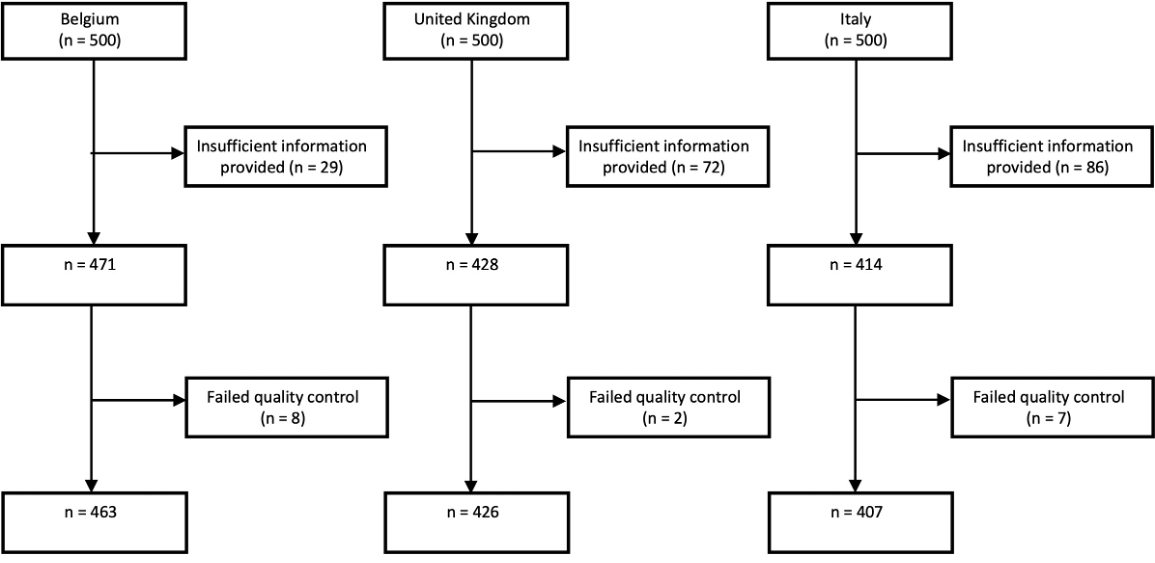

Supplement: Multimedia Appendix 1 [file pediatrics_v8i1e75489_app1.png]
