# Supplementary material for: Exploring Parental Intentions to Use Digital Tools to Enhance Parent-Child Sexual Communication in Europe: Cross-Sectional Questionnaire Study
Source: JMIR Pediatr Parent. 2025 Oct 10;8:e75489. doi: 10.2196/75489 (PMC12552825; doi:10.2196/75489)
Supplement: Multimedia Appendix 2 [file pediatrics_v8i1e75489_app2.docx]

| Item | Mean | SD | ITU1 | ITU2 | ITU3 | PEOU1 | PEOU2 | QT1 | QT2 | QT3 | QT4 | QT5 | QT6 | QT7 | RP1 | RP2 |
| --- | --- | --- | --- | --- | --- | --- | --- | --- | --- | --- | --- | --- | --- | --- | --- | --- |
| ITU1 | 3.37 | 0.86 | 1.00 |  |  |  |  |  |  |  |  |  |  |  |  |  |
| ITU2 | 3.47 | 0.91 | 0.69 | 1.00 |  |  |  |  |  |  |  |  |  |  |  |  |
| ITU3 | 3.53 | 0.83 | 0.67 | 0.64 | 1.00 |  |  |  |  |  |  |  |  |  |  |  |
| PEOU1 | 3.41 | 1.10 | 0.04 | 0.01 | 0.00 | 1.00 |  |  |  |  |  |  |  |  |  |  |
| PEOU2 | 3.58 | 1.16 | 0.01 | -0.04 | -0.02 | 0.83 | 1.00 |  |  |  |  |  |  |  |  |  |
| QT1 | 3.75 | 0.85 | 0.09 | 0.19 | 0.07 | -0.10 | -0.10 | 1.00 |  |  |  |  |  |  |  |  |
| QT2 | 3.94 | 0.73 | 0.31 | 0.33 | 0.29 | -0.02 | -0.04 | 0.46 | 1.00 |  |  |  |  |  |  |  |
| QT3 | 3.87 | 0.75 | 0.35 | 0.40 | 0.33 | 0.01 | -0.02 | 0.37 | 0.61 | 1.00 |  |  |  |  |  |  |
| QT4 | 3.80 | 0.79 | 0.36 | 0.41 | 0.37 | 0.09 | 0.08 | 0.26 | 0.51 | 0.62 | 1.00 |  |  |  |  |  |
| QT5 | 3.56 | 0.88 | 0.41 | 0.46 | 0.39 | 0.04 | 0.00 | 0.26 | 0.39 | 0.43 | 0.55 | 1.00 |  |  |  |  |
| QT6 | 3.96 | 0.70 | 0.37 | 0.43 | 0.37 | 0.02 | 0.02 | 0.31 | 0.52 | 0.54 | 0.56 | 0.46 | 1.00 |  |  |  |
| QT7 | 3.75 | 0.85 | 0.38 | 0.45 | 0.37 | -0.01 | -0.04 | 0.38 | 0.47 | 0.55 | 0.51 | 0.48 | 0.54 | 1.00 |  |  |
| RP1 | 4.27 | 0.76 | 0.18 | 0.17 | 0.15 | 0.07 | 0.06 | 0.17 | 0.26 | 0.24 | 0.21 | 0.16 | 0.26 | 0.22 | 1.00 |  |
| RP2 | 4.18 | 0.76 | 0.19 | 0.20 | 0.2 | 0.05 | 0.03 | 0.19 | 0.23 | 0.24 | 0.20 | 0.20 | 0.28 | 0.23 | 0.71 | 1.00 |
