# Supplementary material for: Exploring Parental Intentions to Use Digital Tools to Enhance Parent-Child Sexual Communication in Europe: Cross-Sectional Questionnaire Study
Source: JMIR Pediatr Parent. 2025 Oct 10;8:e75489. doi: 10.2196/75489 (PMC12552825; doi:10.2196/75489)
Supplement: Multimedia Appendix 3 [file pediatrics_v8i1e75489_app3.docx]

| Items | Standardised factor loading |
| --- | --- |
| *Intention to Use* (ITU) |  |
| ITU1 | 0.84 |
| ITU2 | 0.83 |
| ITU3 | 0.79 |
| *Perceived Ease of Use* (PEOU) |  |
| PEOU1 | 0.94 |
| PEOU2 | 0.89 |
| *Quality of Technology* (QT) |  |
| QT1 | 0.46 |
| QT2 | 0.71 |
| QT3 | 0.78 |
| QT4 | 0.76 |
| QT5 | 0.64 |
| QT6 | 0.73 |
| QT7 | 0.72 |
| *Relevance to Parenting* (RP) |  |
| RP1 | 0.84 |
| RP2 | 0.85 |
