# Supplementary material for: Exploring Parental Intentions to Use Digital Tools to Enhance Parent-Child Sexual Communication in Europe: Cross-Sectional Questionnaire Study
Source: JMIR Pediatr Parent. 2025 Oct 10;8:e75489. doi: 10.2196/75489 (PMC12552825; doi:10.2196/75489)
Supplement: Multimedia Appendix 4 [file pediatrics_v8i1e75489_app4.docx]

| Construct | Unstandardised β | Standardised β | | *P* value |
| --- | --- | --- | --- | --- |
| *Relevance to Parenting (RP) has a direct and positive association with Intention to Use.* | | | | |
| RP → ITU | 0.10 | 0.09 | | 0.002 |
| *QT has a direct and positive association with ITU.* | | | | |
| QT → ITU | 1.09 | 0.59 | | <0.001 |
| *H4: Age has a direct association with Quality of Technology (QT), Relevance to Parenting (RP), and Intention to Use (ITU)* | | | | |
| Age → QT  Age → RP  Age → ITU | 0.00  0.00  -0.01 | -0.01  0.03  -0.06 | | -  -  0.03 |
| *H5: Gender has a direct association with Quality of Technology (QT), Relevance to Parenting (RP), and Intention to Use (ITU)^a^* | | | | |
| Male gender → QT  Male gender → RP  Male gender → ITU | -0.01  -0.20  0.14 | -0.01  -0.15  0.10 | | -  <0.001  <0.001 |
| Other gender identity → QT  Other gender identity → RP  Other gender identity → ITU | 0.09  -0.23  0.15 | 0.04  -0.06  0.04 | | -  0.04  - |
| *H6: Country has a direct association with Quality of Technology (QT), Relevance to Parenting (RP), and Intention to Use (ITU)^b^* | | | | |
| Belgium → QT  Belgium → RP  Belgium → ITU | -0.10  0.04  -0.11 | | -0.13  0.03  -0.07 | <0.001  -  0.02 |
| Italy → QT  Italy → RP  Italy → ITU | -0.03  0.03  -0.02 | | -0.04  0.02  -0.02 | -  -  - |
| *H7: Education level has a direct association with Quality of Technology (QT), Relevance to Parenting (RP), and Intention to Use (ITU)^c^* | | | | |
| Secondary education → QT  Secondary education → RP  Secondary education → ITU | 0.14  0.20  -0.07 | | 0.12  0.10  -0.03 | -  -  - |
| College education → QT  College education → RP  College education → ITU | 0.16  0.33  -0.01 | | 0.21  0.25  -0.01 | -  0.047  - |
| Tertiary or higher education → QT  Tertiary or higher education → RP  College education → ITU | 0.12  0.36  0.01 | | 0.16  0.28  0.01 | -  0.03  - |

Reference groups are ^a^female, ^b^United Kingdom, and ^c^primary education level.
